# [Retracted] MicroRNA-325 inhibits the proliferation and induces the apoptosis of T cell acute lymphoblastic leukemia cells in a BAG2-dependent manner 

**DOI:** 10.3892/etm.2026.13216

**Published:** 2026-06-22

**Authors:** Fengyu Wang, Fengli Wang, Shengyu Zhang, Xiaogang Xu

Exp Ther Med 21:631, 2021; DOI: 10.3892/etm.2021.10063

Following the publication of this paper, it was drawn to the Editor’s attention by a concerned reader that the changes in expression levels of the protein Bax in Jurkat cells reported for the western blot experiments in Fig. 4 were unexpected, since it has been previously been shown for this cell type that they are ‘BAX-null’, and therefore these findings required at least further explanation. In addition, flow cytometric data featured in Fig. 6B on p. 6 were found to be strikingly similar to data which had already been submitted to the same journal (*Experimental and Therapeutic Medicine*) in another paper written by different authors.

In view of the fact that the abovementioned data had already been submitted for publication in an unrelated article prior to the receipt of this paper to *Experimental and Therapeutic Medicine*, the Editor has decided that it should be retracted from the Journal. The authors were asked for an explanation to account for these concerns, but the Editorial Office did not receive a reply. The Editor apologizes to the readership for any inconvenience caused.

